# Factors Requiring Adjustment in the Interpretation of Serum Carcinoembryonic Antigen: A Cross-Sectional Study of 18,131 Healthy Nonsmokers

**DOI:** 10.1155/2017/9858931

**Published:** 2017-05-16

**Authors:** Hae Yeon Kang, Eun Kyung Choe, Kyu Joo Park, Young Lee

**Affiliations:** ^1^Department of Internal Medicine, Healthcare Research Institute, Seoul National University Hospital Healthcare System Gangnam Center, Seoul, Republic of Korea; ^2^Department of Surgery, Healthcare Research Institute, Seoul National University Hospital Healthcare System Gangnam Center, Seoul, Republic of Korea; ^3^Department of Surgery, Seoul National University College of Medicine, Seoul, Republic of Korea; ^4^Healthcare Research Institute, Seoul National University Hospital Healthcare System Gangnam Center, Seoul, Republic of Korea

## Abstract

Serum carcinoembryonic antigen (CEA) is a well-known tumor marker for colorectal adenocarcinoma. However, CEA levels can be influenced by various nonmalignant conditions. A retrospective, cross-sectional study was performed including 18,131 healthy nonsmokers who underwent health check-ups with evaluation of the serum CEA level. In the training set, multivariate analysis revealed that the log-transformed CEA level had positive relationships with age (regression coefficient (*r*) = 0.005, *P* < 0.001), white blood cell (WBC) count (*r* = 0.007, *P* = 0.016), hemoglobin (HB, *r* = 0.016, *P* < 0.001), aspartate aminotransferase (AST, *r* = 0.002, *P* = 0.005), creatinine (*r* = 0.076, *P* = 0.038), and glycosylated hemoglobin (HbA1c, *r* = 0.052, *P* < 0.001); body mass index (BMI, *r* = −0.007, *P* < 0.001) showed a negative correlation. The results for age, BMI, WBC count, HB, AST, and HbA1c were validated in the test set. We were able to construct the following model to predict the log-transformed CEA level: log (CEA + 0.51) = −0.204 − 0.051 (gender) + 0.005 (age) − 0.006 (BMI) + 0.008 (WBC count) + 0.016 (HB) + 0.002 (AST) + 0.062 (creatinine) + 0.054 (HbA1c). For colorectal cancer prediction, the model with the observed CEA and adjusted CEA levels had significantly high predictive power (AUC 0.756, *P* < 0.001) than the model only including the observed CEA level (AUC 0.693, *P* < 0.001). Factors influencing serum CEA levels should be adjusted before clinical interpretation to increase the predictive value of CEA.

## 1. Introduction

Serum carcinoembryonic antigen (CEA), a glycosylated cell surface glycoprotein [1] with reported glycosylation differences between normal and tumor tissues [2], is widely used as a tumor marker. Since it was first identified in 1965 [3], the CEA level has primarily been used to monitor recurrence or to evaluate response to treatment in adenocarcinoma patients [4]. Unfortunately, it is not a disease-specific or tumor-specific marker. Thus, the predictive value of CEA in recurrence surveillance is insufficient [5, 6]. In addition, the screening of serum CEA levels is not recommended in healthy populations because of its low sensitivity and specificity for malignancy, particularly for early stages of colorectal carcinoma (CRC). Moreover, serum CEA levels vary among individuals in healthy populations, and the factors that affect it have not been fully elucidated.

Although there are limitations in CEA measurement, its utility for the surveillance of malignancy recurrence, especially for CRC, is clear. Additionally, the CEA level is being increasingly measured to complement other diagnostic modalities because it is easily performed at a relatively low cost [7]. To improve the precision of CEA measurement in predicting malignancy, the CEA level must be adjusted for confounding factors before interpretation. The purpose of this study was to elucidate the factors that influence the serum CEA level and to improve the accuracy of CEA measurement in a large healthy study population.

## 2. Methods

From January 2013 to December 2013, we performed a retrospective, cross-sectional study on 22,412 people who participated in a routine health check-up program at the Seoul National University Hospital Gangnam Center. We enrolled healthy nonsmokers who had undergone blood sampling to measure the serum CEA level. A total of 18,131 individuals remained after application of the following exclusion criteria: current smoking, a history or presence of gastrointestinal tract malignancy, including CRC or lung disease, an abnormal liver or kidney function profile (i.e., serum aspartate aminotransferase (AST) ≥ 80 IU/L or serum creatinine ≥ 1.4 mg/dL), and acute inflammatory status. We excluded smokers because CEA is well known to be influenced by smoking [8], and different guidelines are required to define the normal CEA level in smokers (nonsmokers, <5 ng/mL versus smokers, <7.5 ng/ mL). Furthermore, it is difficult to quantify the level of smoking in an individual. Blood sampling was performed to evaluate the following: white blood cell (WBC) count; hemoglobin (HB); fasting glucose; lipid profile including total cholesterol, low-density lipoprotein cholesterol (LDL cholesterol), and triglycerides; glycosylated hemoglobin (HbA1c); creatinine; AST; and high sensitivity C-reactive protein (hs-CRP). Venous sampling was performed after 12 hours of overnight fasting. A self-reported questionnaire was completed that included smoking history, current medications, and underlying medical conditions such as hypertension, diabetes, malignancy, and inflammatory disease. Anthropometric measurements, such as height and weight, were performed by a trained nurse using a standardized protocol. For CEA levels of greater than 5 ng/mL (which is an abnormal value, as suggested by the manufacturer), we repeated the test to determine whether any temporary change had occurred. If the level remained greater than 5 ng/mL, a thorough workup, including colonoscopy and low-dose chest CT, was performed to exclude subjects with malignancy.

### 2.1. Ethics Statement

The Institutional Review Board of the Seoul National University Hospital approved the study protocol (IRB number H-1504-027-662), and the study was conducted in accordance with the Declaration of Helsinki. Informed consent was waived by the board.

### 2.2. Serum CEA Measurement

The serum CEA level was measured by an immunoradiometric assay (IRMA) supplied by Radim (Rome, Italy). All of the assays were reported according to the manufacturer's guidelines, and normal levels were <5 ng/mL. All CEA levels below 0.5 ng/mL were reported as <0.5 ng/mL, because the detector was not sensitive enough to determine exact concentrations under that cutoff level. A total of 1937 subjects had a CEA level of below 0.5 ng/mL. In these cases, we arbitrarily assigned a level of 0.49 ng/mL.

### 2.3. Statistics

Continuous variables were expressed as means ± standard deviations (SD). Categorical variables were expressed as frequencies or percentages. The chi-square test for categorical variables and the Student *t*-test or Mann–Whitney *U* test for continuous variables were used to assess the differences between the lowest CEA quartile (Q1) and the highest CEA quartile (Q4) (creatinine, triglycerides, and hs-CRP were evaluated using the Mann–Whitney *U* test). For standardization, the CEA levels were log-transformed, and the value of 0.51 was added to each level to adjust the minimum log-transformed CEA level to zero. Thus, log-transformed CEA was defined as log (CEA level + 0.51). We conducted censored regression analysis to identify factors associated with the log-transformed CEA level. Factors for multivariate analysis were selected using the likelihood ratio test based on the maximum likelihood method. Censored regression analysis was performed because of the arbitrary designation of CEA levels below 0.5 ng/mL as 0.49 ng/mL [9]. Additionally, logistic regression analysis was performed to predict the Q1 versus Q4 CEA level groups. The results were verified using training and validation sets. We randomly selected a training set composed of 50% of the total population. The remaining 50% of the population was used as a test set to check the reproducibility of the results. To identify the discriminatory power between the designed models, we assessed areas under the receiver operating characteristic (ROC) curves. The results were considered statistically significant at a *P* < 0.05. All statistical analyses were performed using SPSS software, version 19.0 (SPSS, Chicago, IL, USA) and R 3.2.2 (R Development Core Team; R Foundation for Statistical Computing, Vienna, Austria).

## 3. Results

### 3.1. Patient Characteristics

The eligible group was composed of 18,131 healthy nonsmokers aged 50.8 ± 11.3 years including 8596 females and 9535 males. The mean serum CEA level was 1.4 ± 0.7 ng/mL in the males and 1.2 ± 0.8 ng/mL in the females.

Table 1 shows the clinical characteristics of the total population and the population separated according to the Q1 versus Q4 serum CEA levels. The CEA quartiles were categorized as follows: 1st quartile (Q1), <0.8 ng/mL; 2nd quartile (Q2), <1.2 ng/mL; 3rd quartile (Q3), <1.6 ng/mL; and 4th quartile (Q4), ≥1.6 ng/mL. Regarding the demographic features, the Q4 CEA group contained a higher proportion of males (64.1 versus 44.0% *P* < 0.001) and was older (mean age 53.8 ± 11.2 versus 48.3 ± 10.9 years, *P* < 0.001). The Q4 CEA group had significantly higher body mass index (BMI), WBC count, HB, fasting glucose, AST, creatinine, triglycerides, and HbA1c than the Q1 CEA group (*P* < 0.05). The levels of total cholesterol, LDL cholesterol, and hs-CRP were not significantly different between the groups (Table 1).

### 3.2. Factors Influencing Log-Transformed CEA Level and Q1 versus Q4 CEA Groups among the Total Population

Table 2 shows the relationships between the log-transformed serum CEA level and other factors, as determined by censored regression analysis. The log-transformed serum CEA level was significantly positively correlated with age (regression coefficient (*r*) = 0.06, *P* < 0.001), BMI (*r* = 0.009, *P* < 0.001), the WBC count (*r* = 0.016, *P* < 0.001), HB (*r* = 0.036, *P* < 0.001), fasting glucose (*r* = 0.003, *P* < 0.001), AST (*r* = 0.005, *P* < 0.001), creatinine (*r* = 0.301, *P* < 0.001), triglyceride (*r* = 0.000, *P* < 0.001), and HbA1c levels (*r* = 0.093, *P* < 0.001). Total cholesterol (*P* = 0.155), LDL cholesterol (*P* = 0.599), and hs-CRP (*P* = 0.523) showed no significant correlation.

We used factors that were significant in the univariate analysis to perform multivariate regression analysis to construct a model for predicting the log-transformed CEA level. Among the total population, age (*r* = 0.005, *P* < 0.001), the WBC count (*r* = 0.008, *P* < 0.001), HB (*r* = 0.016, *P* < 0.001), AST (*r* = 0.002, *P* < 0.001), creatinine (*r* = 0.062, *P* = 0.014), and HbA1c levels (*r* = 0.054, *P* < 0.001) were positively correlated with log-transformed CEA, whereas BMI (*r* = −0.006, *P* < 0.001) displayed a significant negative correlation. Based on this analysis, we were able to construct the following model to predict the log-transformed CEA level:

Log (CEA + 0.51) = −0.204 − 0.051 (gender) + 0.005 (age) − 0.006 (BMI) + 0.008 (WBC count) + 0.016 (HB) + 0.002 (AST) + 0.062 (creatinine) + 0.054 (HbA1c). (Reference for gender was male).

Multivariate analysis was performed to construct a model to predict the Q1 versus Q4 CEA level groups. Age (odds ratio (OR) = 1.037, 95% confidence interval (CI) = 1.033 to 1.042, *P* < 0.001), BMI (OR = 0.957, 95% CI = 0.940 to 0.974, *P* < 0.001), the WBC count (OR = 1.058, 95% CI = 1.024 to 1.093, *P* = 0.001), HB (OR = 1.141, 95% CI = 1.089 to 1.195, *P* < 0.001), AST (OR = 1.011, 95% CI = 1.005 to 1.018, *P* < 0.001), creatinine (OR = 1.540, 95% CI = 1.017 to 2.335, *P* = 0.042), and HbA1c levels (OR = 1.461, 95% CI = 1.329 to 1.611, *P* < 0.001) were significant factors in the model (Table 3).

### 3.3. Validation of the Models for Predicting the Log-Transformed CEA Level and Q1 versus Q4 CEA Groups

In the training set, after adjusting for gender, multivariate regression analysis revealed positive relationships between log-transformed CEA and age (*r* = 0.005, *P* < 0.001), the WBC count (*r* = 0.007, *P* = 0.016), HB (*r* = 0.016, *P* < 0.001), AST (*r* = 0.002, *P* = 0.005), creatinine (*r* = 0.076, *P* = 0.038), and HbA1c levels (*r* = 0.052, *P* < 0.001), whereas BMI (*r* = −0.007, *P* < 0.001) displayed a significant negative correlation. The results for age (*r* = 0.005, *P* < 0.001), BMI (*r* = −0.005, *P* < 0.001), the WBC count (*r* = 0.011, *P* < 0.001), HB (*r* = 0.016, *P* < 0.001), AST (*r* = 0.002, *P* = 0.005), and HbA1c levels (*r* = 0.056, *P* < 0.001) were validated in the test set (Table 2). Multivariate analysis of the model for predicting the Q1 versus Q4 CEA level groups revealed that age (OR = 1.038, 95% CI = 1.031 to 1.044, *P* < 0.001), BMI (OR = 0.949, 95% CI = 0.926 to 0.973, *P* < 0.001), HB (OR = 1.127 95% CI = 1.056 to 1.204, *P* < 0.001), AST (OR = 1.010, 95% CI = 1.001 to 1.020, *P* = 0.025), and HbA1c levels (OR = 1.438, 95% CI = 1.264 to 1.644, *P* < 0.001) were significant factors in the training set. The significance of age (OR = 1.037, 95% CI = 1.030 to 1.043, *P* < 0.001), BMI (OR = 0.964, 95% CI = 0.940 to 0.989, *P* = 0.005), HB (OR = 1.155, 95% CI = 1.081 to 1.235, *P* < 0.001), AST (OR = 1.012, 95% CI = 1.003 to 1.022, *P* = 0.007), and HbA1c levels (OR = 1.484, 95% CI = 1.292 to 1.716, *P* < 0.001) were validated in the test set (Table 3).

To identify the discriminatory accuracy between Q1 and Q4 CEA levels, we calculated the areas under the ROC curves to identify the predictive value of factors selected from this model. The calculated values were 0.684 (*P* < 0.001) for the training set and 0.681 (*P* < 0.001) for the validation set.

### 3.4. Subgroup Analysis in the Normal Population versus the Colorectal Cancer Population with CEA Level between 2 and 4 ng/mL

**Figure 1:**
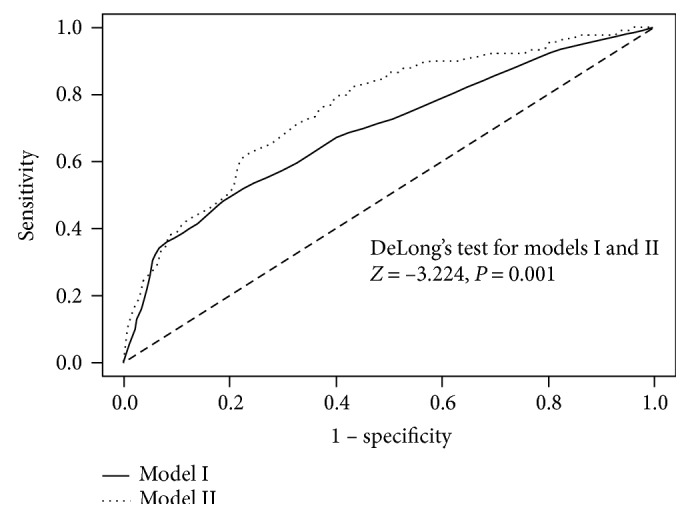
Areas under the curve between control and colorectal cancer individuals with borderline serum CEA levels (2–4 ng/mL). The prediction model is for discriminating between control and colorectal cancer individuals. Model I was designed only with the observed CEA level, whereas model II was designed with the observed CEA level and the adjusted CEA level.

We performed subgroup analysis on patients with a CEA level between 2 and 4 ng/mL, which is a borderline CEA level. Among the eligible population, we included 2364 individuals as the control group and 189 patients with colorectal cancer detected during health check-ups and with a CEA level between 2 and 4 ng/mL as the cancer group. We designed a prediction model for control versus colorectal cancer. One model was designed only with the observed CEA level (model I), and the other model was designed with both the observed CEA level and the adjusted CEA level (model II). We calculated the areas under the ROC curve to compare the discriminative power for the control versus cancer groups of these models. The calculated values were 0.693 (95% CI 0.632–0.754, *P* < 0.001) for model I and 0.756 (95% CI 0.704–0.807, *P* < 0.001) for model II (Figure 1). There were significant differences in AUCs between the two groups according to DeLong's test (*P* = 0.001).

## 4. Discussion

In this study of CEA levels in 18,131 healthy nonsmoking individuals without malignancy or inflammatory disease, factors such as gender, age, the WBC count, BMI, HB, AST, creatinine, and HbA1c levels were found to be significant factors associated with the serum CEA level. This study did not investigate the mechanisms underlying these results; instead, it elucidated the factors influencing CEA comprehensively in a larger population.

CEA is an antigen that is known to be a glycosylated cell surface glycoprotein [1]. It is present not only in a variety of cancers, particularly epithelial tumors, but also in normal mucosal cells [10]. It is used as a tumor marker of malignant lesions in the colorectum, lungs, breasts, and so forth. However, it is also known to be influenced by some benign conditions [11–13]. As a result, measurement of the serum CEA levels is not a standardized screening tool for adenocarcinoma and should not be the only test used to determine the presence or absence of this disease. In clinical practice, the CEA level is mainly used as a marker for follow-up after treatment to monitor tumor recurrence.

At present, CEA measurement is most frequently performed to monitor the recurrence of colorectal cancer after primary treatment. The sensitivity and specificity of CEA in the diagnosis of colorectal cancer have been reported in several papers. For example, Bel et al. reported that the sensitivity of CEA is 37% for patients with Dukes' B stage CRC, 66.6% for those with stage C CRC, and 75% for those with stage D CRC [14]. In a review of various stages of CRC, the sensitivity of CEA has been reported to be 36%, with specificity of 87% for CEA levels >2.5 ng/mL in stage I and II CRC patients [15]. Because of its low sensitivity, measuring CEA levels for the purpose of screening CRC is not recommended. Indeed, CEA has relatively poor sensitivity for predicting locoregional recurrence in the surveillance of CRC recurrence, with a reported rate of less than 60% [16]. Another study evaluating overall recurrence reported sensitivity and specificity ranges of 17–89% and 34–91%, respectively [15]. Several factors contribute to the reduced accuracy of CEA measurement, including heavy smoking [8], glomerular filtration [17], liver function, and chronic inflammation [10–13, 15].

Nevertheless, despite these limitations, CEA is frequently used in clinical practice. Serum CEA measurement is not a standard screening tool for malignancy, but the necessity of its use in medical diagnostics appears to be increasing because it can be easily utilized at a relatively low cost. In fact, CEA measurement is being increasingly performed to complement other cancer diagnostic modules such as colonoscopy, the stool occult blood test, or low-dose chest CT scan. Since Thomson et al. developed a method of measurement in 1969 [18], CEA has been commonly used to monitor recurrence in patients with various gastrointestinal tract cancers and other adenocarcinomas.

Thus, elucidating the factors that affect the CEA level and performing adjustments accordingly might improve the accuracy and utility of its measurement.

In our study, the serum CEA level was positively correlated with the WBC count, HB, AST, and HbA1c levels after adjusting for age, sex, and BMI in multivariate analysis. Previous studies have identified several candidate factors potentially associated with the serum CEA level. The first is metabolic syndrome. Several reports have found that CEA is associated with lipid panel measurements such as LDL cholesterol and triglyceride levels [19]. In our study, neither of these factors showed a significant correlation with CEA after adjusting for age and gender. However, the HbA1c and fasting glucose levels were identified as significant factors, potentially because the lipid profile is also known to be related to BMI and inflammatory status. After adjusting for these factors in the current study, the lipid profile did not exhibit an independent effect on the CEA level. Second, the inflammatory status is potentially related to the CEA level. In our study, we observed that an increased WBC count was positively associated with the CEA level but not with the hs-CRP level. The CEA gene family belongs to the immunoglobulin (Ig) gene superfamily, and expression of the members of this superfamily might reflect the immune status [10, 20]. The WBC count reflects the presence of infection and weakened immunity. However, the CRP level only reflects the acute state of inflammation [21]; therefore, it was not significantly associated with the CEA level. Third, BMI has been suggested to be related to the CEA level. In our study, BMI had a positive relationship with the log-transformed CEA level in univariate analysis and a negative relationship with this level in multivariate analysis. This conflicting result was also observed for the relationship of this level with plasma volume and waist circumference when they were substituted for BMI in the model (data not shown). The results were not verified by analysis of the test set. We assume that BMI is a confounder of multiple factors in the model; consequently, after adjusting for BMI in multivariate analysis, its influence may have been reversed. The negative correlation of the log-transformed CEA level with BMI is in agreement with the results of a previous study examining the relationship between these two factors [22].

Taken together, the results suggest that the CEA level can be misinterpreted under certain circumstances. Underestimation of the CEA level can be critical for CRC patients or other patients with malignancy who are undergoing monitoring for recurrence. In these patients, a decreased HB level or WBC count is often observed because of adjuvant chemotherapy or nutritional deprivation. Thus, adjustments to the CEA level according to the HB level and WBC should precede its interpretation to avoid underestimation. With abnormal liver function or poorly controlled diabetes, the CEA level can be overestimated, which can lead to additional testing that may be unnecessary, during a health check-up or cancer recurrence monitoring. The HbA1c level was validated as a significant factor in the models predicting both the log-transformed CEA levels and Q1 versus Q4 CEA level groups. The number of diabetes patients is increasing, and individuals with this medical condition require adjustments to the CEA level before its interpretation. In the subgroup analysis for those who had a borderline serum CEA level, adding the adjusted CEA level to the observed CEA increased the power for discriminating colorectal cancer (Figure 1). The factors influencing the CEA level that require adjustment should be investigated and validated in a larger malignant population to provide an adjusted formula and cutoff value for clinical practice.

This study has several limitations. First, we did not include the smoking population because of the difficulty with objectively measuring the level of smoking. At our institute, we recommend stopping smoking at least 1 month before a serum CEA level test; nonetheless, those individuals were not included in our study. Because smoking is an important factor influencing the CEA level, it must be investigated in the smoking population after determining objective criteria and constructing a questionnaire to determine smoking levels. The amount and duration of smoking and type of cigarette should be taken into consideration in future studies. Second, because this was a cross-sectional study, we cannot elucidate whether the factors directly affect the CEA level or whether the CEA level is a causal factor affecting the other variables. This should be investigated in a further longitudinal study. Third, there might be other confounding factors that should be included in the model to predict the log-transformed CEA level. However, because the model must be practical for its on-site use, our study only included factors that can be easily and routinely assessed during health check-ups or cancer surveillance. Fourth, we only evaluated the serum CEA level measured by IRMA. CEA belongs to the immunoglobulin gene superfamily, and the CEA subfamily consists of seven expressed proteins, CEA, NCA, BGP, CGM1, CGM2, CGM6, and CGM7 [23]. As sequence homology exists between members of the CEA subfamily, immunological cross-reactivity may occur [23, 24]. Thus, the specificity for measuring CEA could be affected by the selection of antibody. The factor of antibodies should be considered in future analyses.

Our study has some advantages over other reports. First, because all of the included subjects underwent comprehensive medical testing, information about their medical conditions was available, including the history or status of malignancy, inflammation, and underlying disease. Thus, we were able to strictly regulate the inclusion and exclusion criteria for enrollment in the study. Second, the reliability of our analysis is increased because our institute performs more than 20,000 healthy check-ups annually, and the data are stored in electronic medical records.

The results of this study of healthy, nonsmoking individuals suggest that factors such as age, BMI, the WBC count, HB, AST, and HbA1c levels are strongly associated with the serum CEA level. Therefore, these factors should be adjusted before clinical interpretation of the CEA level. The results of this study should be investigated in a larger population of malignancy patients.

## Figures and Tables

**Table 1:** Characteristics of the study population.

	Total population (*N* = 18,131)	CEA Q1 (*N* = 4308)	CEA Q4 (*N* = 4333)	*P* value
Gender				<0.001
Female	8596	2414 (56.0%)	1555 (35.9%)	
Male	9535	1894 (44.0%)	2778 (64.1%)	
Age (years)	50.8 ± 11.3	48.3 ± 10.9	53.8 ± 11.2	<0.001
	51 (17–94)			
Hypertension				<0.001
No		3948 (91.6%)	3735 (86.2%)	
Yes	1947	360 (8.4%)	598 (13.8%)	
Diabetes				<0.001
No		4215 (97.8%)	4096 (94.5%)	
Yes	606	93 (2.2%)	237 (5.5%)	
Body mass index (m^2^/kg)	22.9 ± 3.0	22.7 ± 3.0	23.3 ± 2.9	<0.001
White blood cell count (cells/mL)	5.1 ± 1.4	5.1 ± 1.4	5.9 ± 1.8	0.007
Hemoglobin (g/dL)	14.2 ± 1.4	14.0 ± 1.4	14.5 ± 1.4	<0.001
Fasting glucose (mg/dL)	96 ± 16.5	93.6 ± 13.3	99.3 ± 20.1	<0.001
Total cholesterol (mg/dL)	193.1 ± 33.5	192.7 ± 33.5	193.4 ± 34.5	0.373
AST (IU/L)	22.8 ± 7.4	21.9 ± 7.2	23.9 ± 7.8	<0.001
Creatinine (mg/dL)	0.8 ± 0.2	0.8 ± 0.2	0.9 ± 0.2	<0.001
Triglycerides (mg/dL)	104.4 ± 67.7	98.0 ± 60.4	111.5 ± 72.0	<0.001
LDL cholesterol (mg/dL)	118.7 ± 29.7	119.1 ± 30.0	118.5 ± 30.1	0.336
Hs-CRP	0.1 ± 0.4	0.1 ± 0.4	0.1 ± 0.3	0.349
HbA1c	5.6 ± 0.6	5.5 ± 0.4	5.7 ± 0.7	<0.001

**Table 2:** Univariate and multivariate analyses of log-transformed serum CEA.

	Univariate analysis	Multivariate analysis			
		Total population		Training set	Test set
*N* = 18,131	*P* value	Regression coefficient	*P* value	*P* value	*P* value
Gender (ref.: male)	<0.001	−0.051	<0.001	<0.001	<0.001
Age	<0.001	0.005	<0.001	<0.001	<0.001
BMI	<0.001	−0.006	<0.001	<0.001	<0.001
WBC	<0.001	0.008	<0.001	0.016	<0.001
HB	<0.001	0.016	<0.001	<0.001	<0.001
AST	<0.001	0.002	<0.001	0.005	0.005
Creatinine	<0.001	0.062	0.014	0.038	0.167
HbA1c	<0.001	0.054	<0.001	<0.001	<0.001

**Table 3:** Multivariate analysis of quartile CEA.

	Total population		Training set	Test set
	Odds ratio (95% confidence interval)	*P* value	*P* value	*P* value
Gender (reference: male)	0.668 (0.569–0.784)	0.000	0.000	0.003
Age	1.037 (1.033–1.042)	0.000	0.000	0.000
BMI	0.957 (0.940–0.974)	0.000	0.000	0.005
WBC	1.058 (1.024–1.093)	0.001	0.074	0.003
HB	1.141 (1.089–1.195)	0.000	0.000	0.000
AST	1.011 (1.005–1.018)	0.000	0.025	0.007
Creatinine	1.54 (1.017–2.335)	0.042	0.134	0.162
HbA1c	1.461 (1.329–1.611)	0.000	0.000	0.000

## Additional Points

*Core Tip. *This study showed that CEA levels can vary by body mass index, white blood cell count, hemoglobin, aspartate aminotransferase, creatinine, and glycosylated hemoglobin and that these factors should be adjusted before interpreting CEA levels in clinical practice.
